# Nonsuicidal self-injury: a rapidly evolving global field

**DOI:** 10.1186/s13034-015-0081-4

**Published:** 2015-09-28

**Authors:** Stephen P. Lewis, Paul L. Plener

**Affiliations:** Department of Psychology, University of Guelph, 87 Trent Lane, Guelph, ON N1G 2W1 Canada; Department of Child and Adolescent Psychiatry and Psychotherapy, University Hospital, Ulm, Germany

Since its inception in 2006, the International Society for the Study of Self-injury (ISSS; http://www.itriples.org) has steadily attracted researchers, clinicians, and students from around the globe with a shared interest in better understanding and addressing nonsuicidal self-injury (NSSI). Starting with a meeting in Montreal during the summer of 2006, members of the organization have met annually to disseminate the most current research developments in the field. Pursuant to this initial meeting, ISSS conferences have been hosted in: Ithaca (New York), Boston, Stony Brook (New York), Chicago (twice), New York City, Chapel Hill (North Carolina), and Vancouver.

The 10th annual conference was held in June of 2015 and hosted in Heidelberg, Germany, marking the first time an ISSS meeting was held outside of North America. This not only speaks to the considerable growth of ISSS as an organization but also to the globally recognized importance of the NSSI field. Indeed, since 2006, the number of peer-reviewed papers published about NSSI on an annual basis has almost doubled [1]. Reflecting this explosion of scientific knowledge is the recent inclusion of NSSI as a condition meriting further research in the Diagnostic and Statistical Manual (DSM-5) [2]. Although there is still much to learn, innovations in NSSI research have greatly enhanced our understanding of this important mental health concern.

With researchers attending from four continents and 12 countries, the 2015 ISSS meeting showcased a number of new empirical advances, which illustrate how much the NSSI field has progressed. For instance, this year’s conference included new research examining a number of neurobiological processes implicated in the context of NSSI. In one study, Groschwitz and colleagues used functional magnetic resonance imaging (fMRI) to investigate neural processing of social rejection and physical pain among young people who engaged in NSSI [3]. Findings indicated that youth with elevated depressive symptoms plus a history of NSSI differed from those with just high levels of depressive symptoms and healthy controls in that they had more activation in the medial prefrontal cortex, the anterior insula, and the para-hippocampus when experiencing social exclusion.

Other new lines of research presented at this year’s conference involved experimental procedures in which researchers used in vivo, lab-based proxies for NSSI [4–6]. For example, drawing on methods in pain research, one study used a cold-pressor task, an ethical experimental pain induction task in which participants immerse a forearm into a bath of cold water. In this study, the researchers found that youth with a NSSI history demonstrated a significant increase in vagal activity pursuant to pain induction compared to healthy controls [4]. Collectively, efforts involving lab-based proxies for NSSI are allowing researchers bridge the gap between both psychological and physiological theories to better understand NSSI.

Finally, other highlights from the 2015 ISSS meeting build on research [7] examining the DSM-5 criteria for NSSI Disorder (2). Brausch and Muelhenkamp presented new findings comparing criterion B (pertinent to NSSI functions) of the proposed DSM-5 between individuals who met criteria for NSSI Disorder and those who did not [8]. Aligning with recent studies [8], their results suggested that, individuals reporting more frequent (>5) and more recent (<12 months) NSSI find interpersonal reasons for NSSI to be less relevant. Thus, if criterion B is to be retained, the concluded that emphasis may need to be placed on intrapersonal processes as the most salient reasons for NSSI enactment. Moreover, these findings further underscore the import of and need for research to carefully study the sensitivity and specificity of DSM-5 criteria for NSSI.

Taken together, the aforementioned studies, as well as the other studies presented at the 2015 ISSS meeting, illustrate the rapid evolution of the NSSI field. They also accentuate the need to continue exploring novel ways to better understand (a) the processes involved in NSSI and (b) broader-based research issues (e.g., elucidating valid and clinically useful diagnostic criteria) and related empirical considerations (e.g., ethical concerns, methodological issues). The studies and reviews presented in our second part of the NSSI thematic issue therefore span a broad range of topics focusing both on specific aspects of NSSI as well as on the “big picture”. Research among NSSI among different populations is ever-growing, which raises ethical concerns as NSSI research tends to ask sensitive questions in often vulnerable populations. A working group of the International Society for the Study of Self-injury (ISSS) consisting of authors from three countries, has taken great lengths to provide a thorough review on ethical issues associated with NSSI research. This position paper by Lloyd-Richardson et al. goes far beyond a review of ethical challenges, such as questions of consent, assent and confidentiality, as it provides guidelines for ethical sensitive research in NSSI and also raises new questions for upcoming research in NSSI. The paper also puts a focus on the aspect of iatrogenic risks stemming from NSSI research and presents strategies to mitigate risk. Complimenting this paper is research from a large Australian longitudinal community sample, which provides empirical data about ethical aspects of NSSI research participation. Hasking et al. were able to show, that the great majority (74 %) of participants enjoyed taking part in NSSI related research, while only 5 % reported a negative experience associated with research participation.

As the DSM-5 has introduced diagnostic criteria for NSI in Section 3 as a “condition for further study”, more and more studies have used this criteria to operationalize NSSI. Maria Zetterqvist presents an overview on 16 empirical studies using the DSM-5 diagnostic criteria, reporting that people fulfilling criteria for DSM-5 NSSI disorder are among the most severely impaired individuals on the spectrum of people with a history of NSSI. However, endorsement rates for the different criteria of the DSM-5 diagnosis seem to vary across studies, with higher endorsement rates for negative states or interpersonal difficulties preceding NSSI. Following up on the functions of NSSI, Klonsky et al. present a study on two large patient samples, in which functions of NSSI were assessed. The researchers found evidence of two main functions: intrapersonal (e.g., affect regulation and anti-dissociation) as well as social functions. The review by Arbuthnott and Lewis shifts focus from individuals engaged in NSSI to those who are often involved in the supporting those who struggle with NSSI, namely: parents of youth who self-injure. In the 82 papers that were reviewed, parents are described as possible sources of support for adolescents who self-injure and who are involved in therapeutic interventions. However, the authors note that parent wellbeing can be compromised as part of the process of supporting their children. Hence, clinicians should be aware of the impact of NSSI on caregivers and should encourage those parents to engage in self-care.

Targeting specific themes, several new studies on different aspects of NSSI are included in this special issue: In the study from Trepal et al. data are presented from a college student sample with a particular focus on coping strategies. Students who report recent NSSI showed higher levels of maladaptive coping strategies and lower levels of ethnic belonging. Following up on the question of ethnic belonging more specifically, Plener et al. take a closer look on immigration as risk factors for both NSSI and suicide attempts in an adolescent community sample. Suicidality is also the focus of two other studies presented in this issue. In a study by Tanner et al. involving 77 adolescents who engaged in NSSI and fire-setting, youth who reported a suicide attempt in the past had higher rates of victimization and more severe NSSI. Finally, in a study from Koenig at el findings from a large community sample of adolescents in Germany are presented. Recurrent idiopathic pain was associated with both NSSI and suicide attempts, leaving the authors to conclude that adolescents with recurrent pain should be evaluated for NSSI and suicidality.
